# The Beijing Sentence Corpus II: A cross-script comparison between traditional and simplified Chinese sentence reading

**DOI:** 10.3758/s13428-024-02523-z

**Published:** 2025-01-17

**Authors:** Ming Yan, Jinger Pan, Reinhold Kliegl

**Affiliations:** 1 Department of Psychology, University of Macau, Macau, Macao; 2 Center for Cognitive and Brain Sciences, University of Macau, Macau, Macao; 3 Department of Psychology, The Education University of Hong Kong, Ting Kok, Hong Kong; 4 Department of Sport and Health Sciences, University of Potsdam, Potsdam, Germany

**Keywords:** Eye movement corpus, Traditional Chinese, Simplified Chinese, Reading

## Abstract

We introduce a sentence corpus with eye-movement data in traditional Chinese (TC), based on the original Beijing Sentence Corpus (BSC) in simplified Chinese (SC). The most noticeable difference between TC and SC character sets is their visual complexity. There are reaction time corpora in isolated TC character/word lexical decision and naming tasks. However, up to now natural TC sentence reading corpus with recorded eye movements has not been available for general public. We report effects of word frequency, visual complexity, and predictability on eye movements on fixation location and duration based on 60 native TC readers. In addition, because the current BSC-II sentences are nearly identical to the original BSC sentences, we report similarities and differences of the linguistic influences on eye movements for the two varieties of written Chinese. The results shed light on how visual complexity affects eye movements. Together, the two sentence corpora comprise a useful tool to establish cross-script similarities and differences in TC and SC.

Eye movements in reading represent a complex dynamic system influenced by visual, lexical, and syntactic processes of previous, present, and upcoming words. Eye movement control during reading involves two decisions, including a spatial decision about where to send the eyes, as measured in fixation location (FL, landing position of the first fixation on a word relative to word beginning), as well as a temporal decision about when to move the eyes, as measured in fixation duration (e.g., Brysbaert, et al., 2005; Vainio et al., 2009). Much evidence about eye movements in reading has been obtained from cross-language studies. Due to its logographic nature and other fundamental differences from alphabetic scripts, the Chinese writing system has provided several theoretical challenges to theories of eye movement control in reading. For instance, readers can typically obtain useful information within each fixation from a larger area beyond the currently fixated foveal word, known as the perceptual span, which covers 14–15 upcoming letters for English reading (McConkie & Rayner, 1975). Presumably due to higher text density in Chinese than English, the perceptual span is physically smaller, including up to four upcoming characters for Chinese (e.g., Inhoff & Liu, 1998; Yan et al., 2015). Another unique feature of the Chinese orthography is its direct representation of meaning (Hoosain, 1991; Yan & Kliegl, 2023), evident by fast semantic access while bypassing phonological mediation (Chen & Shu, 2001; Zhou & Marslen-Wilson, 1999, 2000). Such a language-specific characteristic led to early semantic activation from parafoveal words before fixated upon (e.g., Yan et al., 2009), whereas such effects have been traditionally elusive in English for decades (e.g., Inhoff, 1982; Inhoff & Rayner, 1980; Rayner et al., 1986), arguably because phonology activation is known to precede semantic activation in alphabetic scripts (van Orden, 1987). These examples demonstrate that cross-language comparisons of eye movements in reading promote our knowledge about language-universal and specific visual, cognitive, and oculomotor processes, which in the long run contribute to the development of a complete and realistic model of eye-movement control during text reading. In this article, we first review relevant features of the Chinese writing system, then introduce a traditional Chinese (TC) sentence corpus with eye-movement data. Finally, combining a previously reported simplified Chinese (SC) dataset, which contains nearly identical sentences as the current TC corpus, we provide fixation location and duration analyses, highlighting that the two varieties of the Chinese writing system are substantially similar with respect to the spatial and temporal decisions of eye movements in reading.

The Chinese characters vary in their number of strokes and take the same squared space. As such, visual complexity increases in case of a character with more strokes. The first standardized character set, the small seal script, has been adopted as the official script of China in the Qin dynasty (221 – 206 BC). The shapes of modern characters first appeared with the clerical script in early Han dynasty, dated back to 200 BC. These characters look highly similar to the current written Chinese characters. Because they preserve the visual shapes of ancient characters, they are commonly referred to as *traditional Chinese* characters, which are officially used in many Chinese-speaking regions, such as Taiwan, Hong Kong, and Macau. In contrast, another writing variation of the script, *simplified Chinese,* has been used officially since the 1950s in mainland China. The simplification included not only reduction of strokes while retaining visual contour (e.g., simplified 爱 for traditional 愛, *love*), but also structural simplification (e.g., simplified 亲 for traditional 親, *parent*) and merging different TC characters into a single SC character (e.g., simplified 面 for traditional characters 麵, *flour*, and 面, *face*). Apparently, all the simplification principles above have reduced the number of strokes of the original TC characters. The simplification procedure has influenced approximately 40% of all Chinese characters, while the rest are identical between TC and SC (e.g., 偷, *steal*).

Although most current eye-tracking studies on Chinese reading adopted SC as their reading materials, it is interesting to notice that research on the language in fact began with TC. The first studies on eye movements in Chinese reading (Chen & Carr, 1926; Shen, 1927) have established that basic oculomotor behaviors when reading logographic Chinese, despite its lack of inter-word spaces, are fundamentally like alphabetic scripts such as English. These studies were conducted long before SC was officially introduced. Other studies on TC have shown its similarity in oculomotor activities with SC. For instance, studies have shown that TC readers can obtain visual, phonological, and morpho-semantic knowledge from parafoveal words (Tsai et al., 2004, 2012; Yen et al., 2008, 2009).

As reviewed above, studies on TC reading have adopted a (quasi-)experimental approach, orthogonally manipulating only one or two factors. The corpus-analytic approach, on the other hand, is based on much larger samples of observations and simultaneously evaluates influences of more variables related to the reading process. This allows corpus analyses to cover much wider and more complete ranges of independent variables than orthogonally designed factors, which typically include only a limited number of conditions. In this case, researchers benefit from the corpus-analytic approach in observing more reliable and comprehensive trends of the independent variables. To this end, reading and eye movement corpora have provided milestones results (Just & Carpenter, 1980; Rayner, 2009). In general, influences of word frequency, predictability, and length of the current word on fixation duration have been well documented and are considered the most robust predictors of oculomotor indices. Words that appear more often in a language, are shorter in length, and can be guessed more easily from prior contexts are typically fixated on more briefly than their less frequent, longer, and less predictable counterparts (e.g., Kliegl et al., 2004; Kuperman & Van Dyke, 2011). Considering that reading can also be influenced by neighbor words, the Potsdam Sentence Corpus (PSC; Kliegl, 2007; Kliegl et al., 2004, 2006) additionally included the corresponding properties of words N-1 and N + 1 as model predictors and conducted more comprehensive analyses, showing that in natural sentence reading, lexical processing of the fixated word is simultaneously influenced by word properties of the past, present, and future words.

Eye-tracking corpora are broadly available for many scripts including Dutch (Cop et al., 2017), English (Schilling et al., 1998), French (Kennedy & Pynte, 2005), German (Kliegl et al., 2006), Hindi (Husain et al., 2014), Russian (Laurinavichyute et al., 2019), Turkish (Özkan et al., 2021), Uighur (Yan et al., 2014), etc. Although there have been databases for TC character and word lexical decision and naming reaction time (Chang et al., 2016; Tsang et al., 2024; Tse et al., 2017, 2022), all existing corpora for sentences and eye movements are in SC (Li et al., 2014; Pan et al., 2022a; Yan et al., 2019a). The database that is most relevant to the present study was conducted by Tsang et al. (2024), who explored how visual complexity influences written word recognition from a cross-script perspective by comparing SC and TC lexical decision reaction times. With large samples of participants and Chinese words, their results showed stronger effects of word frequency, word length, and number of strokes in SC than in TC, concluding SC readers focus more on local properties of the words than TC readers. The simplification in SC, with a reduced number of strokes on average, renders SC characters less distinguishable than TC characters (e.g., characters 農, *agriculture*, and 衣, *clothes*, are visually distinctive in TC, whereas their simplified writings 农 and 衣 are very similar). Therefore, it was proposed that SC readers acquire more vocabulary knowledge and develop better visual-perceptual skills than TC readers, as they need to distinguish characters on the basis of fewer distinctive visual features in SC (McBride-Chang et al., 2005).

Nevertheless, cautions must be made concerning several possible methodological issues with a lexical decision or naming task. Although reaction time to single words provides valuable information on momentary lexical processes, words seldom show up in isolation in daily life. Instead, they usually emerge in phrases, sentences, and passages. During the reading of continuously written text, readers’ perceptual span covers an area much further beyond the currently fixated word. In other words, visual and lexical processes of a word start when it is in the parafovea, that is, before it is fixated upon. Thus, presenting an isolated single word in the foveal vision is not only unnatural, but also deprives its early parafoveal processing. Moreover, natural reading of sentences involves not only progressive saccades to a next word, but also allows readers to skip and refixate on certain words and to regress back to a word that has been previously read. These complete dynamics of eye movements are difficult to capture when analyzing reaction time in lexical decision or naming tasks or focusing on a small number of target words in orthogonal experimental designs.

In the current study, we report an eye-movement corpus for sentence reading in TC.^1^ Sentences written in SC and TC can be identical except for their visual difference. Therefore, we adopted the Beijing Sentence Corpus (BSC; Pan et al., 2022a; Yan et al., 2010), which was created originally in SC, and converted the sentences to TC. There are two major advantages to build a TC corpus based on BSC. First, nearly all sentences and words were identical to the original BSC in SC, except for some minor vocabulary differences between mainland China (SC) and Macau (TC), which we elaborate on in detail below. This allows for a within-item cross-script comparison between SC and TC. Additionally, we make available predictability norms that have already been collected in SC, allowing for evaluations of predictability effects of the past, current, and future words on fixation location and duration during TC sentence reading. As introduced earlier, predictability effect is one of the most important factors in predicting eye movements in reading. However, traditionally, it takes tremendous amount of time and effort to collect reliable predictability norms for words in sentence corpora, because word predictabilities are evaluated using the cloze test^2^: Participants are presented with sentence frames prior to target words and are instructed to guess the missing words and complete the sentences (Taylor, 1953). Therefore, only a few existing eye-movement corpora have included predictability information (e.g., Kennedy et al., 2013; Luke & Christianson, 2016). The PSC is a corpus with complete predictability norms for all words and has evaluated the norms with a large sample of readers (i.e., 272 native German speakers, yielding 83 predications for each word; Kliegl et al., 2004, 2006). With the reliable predictability norms, Kliegl et al. (2006) demonstrated opposite trends of predictability effects of the current word (N) and the future word (N + 1) in fixation duration on word N. A more predictable word N implies easier lexical processing and, as expected, reduced local fixation duration. Interestingly, a highly predictable word N + 1 increased fixation duration on word N. Likely, memory retrieval of word N + 1 brings forward part of its lexical processing to an earlier temporal stage during fixation on word N and prolongs fixation duration on word N. In contrast, some studies have demonstrated a negative successor predictability effect despite the non-presence of word N + 1 in the parafovea, suggesting that the effect could be due to a predictive process of an upcoming word instead of parafoveal processing (Angele et al., 2015, 2016; see van Schijndel & Schuler, 2016, for similar findings in a self-paced reading task). Li et al. (2014) also reported that highly predictable words N + 1 shortened fixation durations on words N in Chinese, arguably due to parafoveal processing. In sum, the direction of the word N + 1 predictability effect is not consistent in the existing literature and the current study also aimed to shed new light on this question.

In addition to fixation duration, predictability has also been shown to influence fixation location. Pan et al. (2022a) collected predictability norms of all words from the BSC from 148 university students, with 74 guesses for each word, which is roughly comparable to the PSC. They reported positive trends of word N-1 and word N predictabilities on fixation location on word N during SC sentence reading. Note that, saccades towards words N are programmed during fixations on words N-1. Therefore, word N-1 effects are interpreted as foveal effects and word N effects are parafoveal-on-foveal effects for saccade generation. Fixations landed on words N were closer to the word centers when launch words N-1 were more predictable. Arguably, more-predictable launch (foveal) words N-1 free up more attentional resources for better parafoveal processing and saccade targeting. In the meanwhile, fixations on words N also landed further for more-predictable target (parafoveal) words N, possibly because more-predictable words N are easier to be segmented for saccades (Yan et al., 2010).

## Method

### Participants

Sixty students from University of Macau (*M*_*age*_ = 20.5 years, *SD* = 2.7, 32 females and 28 males) with normal or corrected-to-normal vision participated in the eye-tracking experiment. All participants had undertaken their education in Macau (where TC, but not SC, is the official written language and the medium of instruction) since primary school. Experimental procedures were approved by the Ethics Committee of the Department of Psychology, University of Macau (SONA-2023–05). Participants gave their written informed consent prior to the experiment. The sample size was identical to the original BSC (Pan et al., 2022a) and was supported by recent simulation work by Kumle et al. (2021).

### Material

The current reading materials, consisting of 150 TC sentences, were based on the BSC which was created in SC (Pan et al., 2022a; Yan et al., 2010). Words in the BSC were defined by entries in the Modern Chinese Word Frequency Dictionary (Institute of Linguistics Studies, 1986) and the word boundary agreement was rated by 20 native Chinese participants, resulting in a high level of word boundary agreement (i.e., 97%; Yan et al., 2010). The BSC sentences have been modified in the following steps. First, all sentences were converted from SC to TC. Second, we replaced 15 words from mainland Chinese vocabulary by Macau vocabulary with the same length, most of which had identical, similar, or related meanings. For instance, words 伊始 (beginning), 公安局 (police department), 领导 (leaders), 利税 (profit and tax) from the original BSC were replaced by 開始 (beginning), 警察局 (police department), 官員 (officials), 利潤 (profit), respectively. The sentences were on average 21.0 characters (*SD* = 2.5, range: 15 to 25) or 11.2 words (*SD* = 1.6, range: 7 to 15) in length.

### Apparatus

Participants’ eye-movements were recorded with an Eyelink1000 system running at 1000 Hz. Each sentence was presented in a single line on a 24-inch BenQ ZOWIE XL2546K monitor using the Song font (resolution: 1920 × 1080 pixels; frame rate: 240 Hz). The participants were seated comfortably with their heads placed on a chin-and-forehead rest at 70 cm from the monitor. Each character subtended 1.1 degrees of visual angle. All recordings and calibrations were performed monocularly based on the participants’ right eyes and viewing was binocular.

### Procedure

The participants’ gaze-positions were calibrated with a standard 5-point grid (error < 0.5°). After validation of the calibration accuracy, a fixation-target appeared on the left side of the monitor for a drift check. If the eye tracker identified a participant’s gaze on the fixation-target, the fixation-target disappeared and a sentence appeared, with the center of the first character in the sentence presented at the fixation-target position. Otherwise, failure to detect a participant’s gaze on the initial fixation-target initiated a re-calibration. The participants were instructed to read the sentences silently for comprehension, then fixate on a dot in the lower-right corner of the monitor, and finally press a keyboard button to signal trial completion. They started the experiment with 15 warm-up trials, which were not included in analyses reported below. Each participant received the 150 experimental sentences in a different, randomized order, 40 of which were followed by easy yes–no comprehension questions to encourage the participants’ engagement with the reading task. They on average correctly answered 88.6% of them (*SD* = 5.5%).

### Data analysis

Fixations were determined with an algorithm for saccade-detection (Engbert & Kliegl, 2003). Sentences were excluded for containing missing samples, tracker errors, or participants’ blinks or coughs (n = 1057, 11.7%). Table 1 summarizes basic information we have included in the data files, based on which oculomotor indices can be computed. Typically, words with extremely short or long fixations are excluded from data analyses and the criteria may differ among researchers. Therefore, we kept all observations in the data file so that readers can apply their own fixation selection criteria.

**Table 1:** Eye-movement data variables and descriptions

Column	Variable	Mean (SD)	Description
1	ID	\	A unique number for each participant
2	SN	\	A unique number for each sentence
3	NW	\	The total number of words in the current sentence
4	WN	\	The ordinal position of the current word within the sentence
5	FL	0.95 (0.65)	Fixation location relative to word beginning in percent of word length
6	DUR	254.30 (118.95)	Fixation duration in milliseconds
7	AO	−0.01 (437.85)	Outgoing saccade amplitude from the current fixation in number of characters
8	DIR	0.64 (0.77)	Direction of the outgoing saccade
9	O	−0.01 (437.85)	Incoming saccade amplitude from previous fixation
10	L	0.53 (0.16)	Word length, reciprocal of number of characters
11	F	1.51 (1.23)	Word frequency, log-transformed occurrence per million words
12	P	−1.30 (0.67)	Logit predictability of the word
13	I	19.77 (7.98)	Visual complexity, number of strokes of the word
14	WN1	\	The ordinal position of the last word
15	L1	0.61 (0.23)	Word length of the last word
16	F1	2.01 (1.46)	Word frequency of the last word
17	P1	−1.17 (0.77)	Logit predictability of the last word
18	I1	17.08 (8.12)	Visual complexity of the last word
19	WN2	\	The ordinal position of the next word
20	L2	0.60 (0.22)	Word length of the next word
21	F2	1.90 (1.45)	Word frequency of the next word
22	P2	−1.11 (0.82)	Logit predictability of the next word
23	I2	17.83 (8.43)	Visual complexity of the next word
24	WNx1	\	The ordinal position of the last fixated word
25	FLx1	0.96 (0.65)	Fixation location on the last fixated word
26	DURx1	253.72 (114.00)	Fixation duration on the last fixated word
27	AOx1	1.86 (3.40)	Outgoing saccade amplitude from the last fixation
28	DIRx1	0.72 (0.69)	Direction of the last outgoing saccade
29	Ox1	−0.09 (456.20)	Incoming saccade amplitude to the last fixation
30	Lx1	0.53 (0.16)	Word length of the last fixated word
31	Fx1	1.51 (1.22)	Word frequency of the last fixated word
32	Px1	−1.35 (0.77)	Logit predictability of the last fixated word
33	Ix1	19.74 (7.98)	Visual complexity of the last fixated word
34	WNx2	\	The ordinal position of the next fixated word
35	FLx2	0.99 (0.66)	Fixation location on the next fixated word
36	DURx2	256.17 (117.72)	Fixation duration on the next fixated word
37	AOx2	−0.43 (465.33)	Outgoing saccade amplitude from the next fixation
38	DIRx2	0.62 (0.78)	Direction of the next outgoing saccade
39	Ox2	1.86 (3.40)	Incoming saccade amplitude to the next fixation
40	Lx2	0.53 (0.17)	Word length of the next fixated word
41	Fx2	1.49 (1.23)	Word frequency of the next fixated word
42	Px2	−1.28 (0.70)	Logit predictability of the next fixated word
43	Ix2	19.92 (8.11)	Visual complexity of the next fixated word

In corpus analyses, predictors are often correlated with each other. In this case, zero-order regression may be influenced by uncontrolled confounding variables and fails to reveal a true effect. To overcome this problem, linear mixed-effects models (LMMs) have been proven a useful statistical tool to estimate influences of correlated predictors, as well as individual differences at the levels of participants, words, and sentences. As such, partial effects in LMMs (i.e., estimates after statistical control of confounding variables) reveal effects of interest in corpus analyses (Kliegl et al., 2006). Following classic work on preferred viewing location (PVL; McConkie et al., 1988; Rayner, 1979) and applying LMMs to natural reading of SC sentences, Pan et al. (2022a) simultaneously modelled oculomotor, visual, lexical, and sentence-level effects on FL. We report below FL in single-fixation cases, single-fixation duration (SFD; the duration of the fixation on a word when it is fixated on exactly once during first-pass reading) and gaze duration (GD; the cumulative duration of all fixations during the first pass reading of the word) analyses and compared them with the SC data.

Estimates were based on LMMs using the *lme4* package (Version 1.1–23; Bates et al., 2015) and the *lmerTest* package (Version 3.1–2; Kuznetsova et al., 2017) in the R-language environment (R Development Core Team, 2018). The main effects of the FL model included word length, frequency, stroke count and predictability of words N-1 and N, as well as skipping of word N-1 and linear and square trends of launch site. The main effects of the SFD and GD models included the same linguistic properties of words N-1, N and N + 1. The interaction terms of all models included two-covariate interactions. We started with the base-models described above and removed non-significant fixed-effect interaction terms. For random effects, we initially included subject- and item-related variance components for intercepts and random-slopes for the fixed effects. Following a standard parsimonious model selection procedure, correlation parameters and small variance parameters were dropped for successful model convergence (Matuschek et al., 2017).

## Results and discussion

### Fixation location

The FL analyses were motivated by debates concerning saccade generation in Chinese reading. In alphabetic scripts, readers are known to generate saccade towards word centers, where word processing is assumed to be optimal (e.g., McConkie et al., 1989; O’Regan & Lévy-Schoen, 1987). Such word-based saccade-targeting mechanism has been implemented as one of the core principles in computational models of eye movements in reading (e.g., Engbert et al., 2005; Reichle et al., 1998). However, given that low spatial frequency information in the parafoveal and peripheral visual fields are the most reliable information to determine word boundaries for word-based saccades, the lack of inter-word spaces in Chinese has apparently raised a challenge to the current saccade generation theories. Indeed, there has been theoretical debate on whether saccade-targeting in Chinese is word-based or character-based (e.g., Liu et al., 2015; Tsai & McConkie, 2003; Yang & McConkie, 1999).

Yan et al. (2010), proposed that Chinese readers could dynamically switch between a word-based saccade-targeting aiming at the word center and a character-based saccade-targeting aiming at the first upcoming character beyond the currently fixated word, contingent on their successfulness in segmenting the saccade-target word. By using predefined word boundary knowledge as a model input to simulate eye movements during Chinese sentence reading, Rayner et al. (2007) achieved good simulation results. Their work reflected the importance of word segmentation from another perspective, although provided little knowledge on *how* strings of unspaced characters are segmented into word units. 

**Fig. 1:**
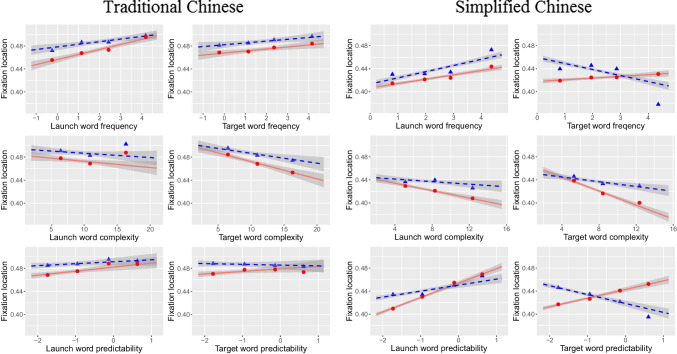
Partial effects (solid lines) of LMMs and zero-order smooths of observed values (dashed lines) of first-fixation location relative to target word length as a function of launch-word and target-word properties for TC (left panel) and SC (right panel) reading. Shaded bands for partial effects represent 95% CIs based on observation-level LMM residuals

Replicating patterns observed during SC reading (Pan et al., 2022a), the current TC data also showed foveal influences of launch-words’ frequency, visual complexity, and predictability on FL (Table 2 and Fig. 1). FL shifted further into the word when the launch word was more frequent, visually less complex, and more predictable. The results may suggest that low foveal processing load frees up more attentional resources for readers’ parafoveal processing (e.g., Henderson & Ferreira, 1990; Hohenstein et al., 2017; Inhoff et al., 2000; Inhoff & Rayner, 1986; Wotschak & Kliegl, 2013) and therefore increases the likelihood of successful parafoveal word segmentation. For influences of parafoveal target-word properties in TC, with a decrease of parafoveal target-words complexity, FL shifted closer to the word centers, indicating that visually simple target-words are easier to be segmented parafoveally for clear word boundaries. The influences of target-word frequency and predictability on FL, however, were not statistically significant. Given the high information density in Chinese texts as compared to alphabetic texts, effects on FL appear to be small in Chinese. Nevertheless, they are important in illustrating influences of linguistic properties on saccade generation.

**Table 2:** Linear mixed model estimates for word N fixation location

		**TC**			**SC**	
**Fixed effect**	**Est**	**SE**	**t**	**Est**	**SE**	**t**
(Intercept)	−0.027	0.008	−3.502	−0.076	0.010	−7.258
k1	0.027	0.010	2.664	0.036	0.009	3.989
poly(lls, 2)1	−17.284	1.033	−16.732	−35.725	0.826	−43.267
poly(lls, 2)2	4.384	0.654	6.699	2.721	0.465	5.858
l1.c	0.016	0.005	3.142	−0.006	0.004	−1.485
f1.c	0.009	0.002	4.367	0.008	0.002	3.881
lgs1.c	−0.009	0.004	−2.053	−0.017	0.004	−4.791
p1.c	0.007	0.003	2.521	0.025	0.003	8.580
l.c	−0.021	0.005	−3.929	−0.026	0.006	−4.302
f.c	0.004	0.002	1.545	0.003	0.002	1.373
lgs.c	−0.020	0.005	−3.643	−0.035	0.005	−6.827
p.c	0.004	0.003	1.542	0.015	0.003	5.360
k1:poly(lls, 2)1	9.172	1.924	4.767	2.971	1.821	1.631
k1:poly(lls, 2)2	3.281	1.343	2.444	15.914	0.864	18.412
k1:l1.c	NA	NA	NA	−0.064	0.008	−7.749
**Variance component**		**SD**			**SD**	
Word—(Intercept)		0.026			0.035	
Sent—(Intercept)		NA			0.013	
Subj—p.c		NA			0.014	
Subj—lgs.c		0.024			0.018	
Subj—f.c		NA			0.004	
Subj—l.c		NA			0.032	
Subj—p1.c		NA			0.015	
Subj—lgs1.c		0.018			0.014	
Subj—f1.c		NA			0.009	
Subj—(Intercept)		0.046			0.070	
Residual		0.252			0.183	
Log-likelihood		−1327.1			9023.3	
Deviance		2654.2			−18,046.6	
AIC		2692.2			−17,980.6	
BIC		2847.5			−17,701.6	
N		26,097			34,689	

k1 = skipping of word n-1, lls = launch site, l1.c = length of word n-1, f1.c = frequency of word n-1, lgs1.c = log stroke count of word n-1, p1.c = predictability of word n-1, l.c = length of word n, f.c = frequency of word n, lgs.c = log stroke count of word n, p.c = predictability of word n, Subj = subject-related variance component, Word = word-(token-)related variance component, Sent = sentence-related variance component

The absence of explicit word boundary, and its consequence of word boundary disagreement, are among the most distinctive features of Chinese script. Indeed, previous studies have considerable debates on saccade generation in Chinese (e.g., Inhoff & Wu, 2005; Liu et al., 2015; Tsai & McConkie, 2003; Yan et al., 2010). These accounts differ with respect to whether saccade generation can be influenced by parafoveal word boundary information. However, existing studies have either adopted an experimental control approach and manipulated a relatively small number of items, or used sentence corpora with clear word boundaries. As such, further investigations are desirable for ecologically valid and generalizable findings. To this end, the Multilingual Eye-movement Corpus (MECO) project compares fixation and saccade behaviors across multiple languages through eye-tracking data during natural passage reading (Siegelman et al., 2022). We believe the project potentially offers the best insights into saccade generation, once it extends to the reading of Chinese and other unspaced scripts.

Additionally, statistical knowledge seems to play a critical role for Chinese word segmentation. For instance, Yen et al. (2012) proposed that character positional probability, that is, the likelihood of a character in different within-word positions, can influence such a character-to-word assignment. A clear word boundary is perceived when a word begins with a character that appears frequently as the first character of words, and when a word ends with a character that locates often as the final character of words (see Reilly et al., 2011 for similar findings in Thai, an alphabetic unspaced script). In a similar vein, Chinese readers showed a processing cost when the two middle characters in two successive two-character words made a highly frequent word, arguing against a strictly serial character-to-word assignment and suggesting that word boundary overlap may blur parafoveal and foveal processing (Inhoff & Wu, 2005; Yan & Kliegl 2016).

### Single-fixation duration

**Fig. 2:**
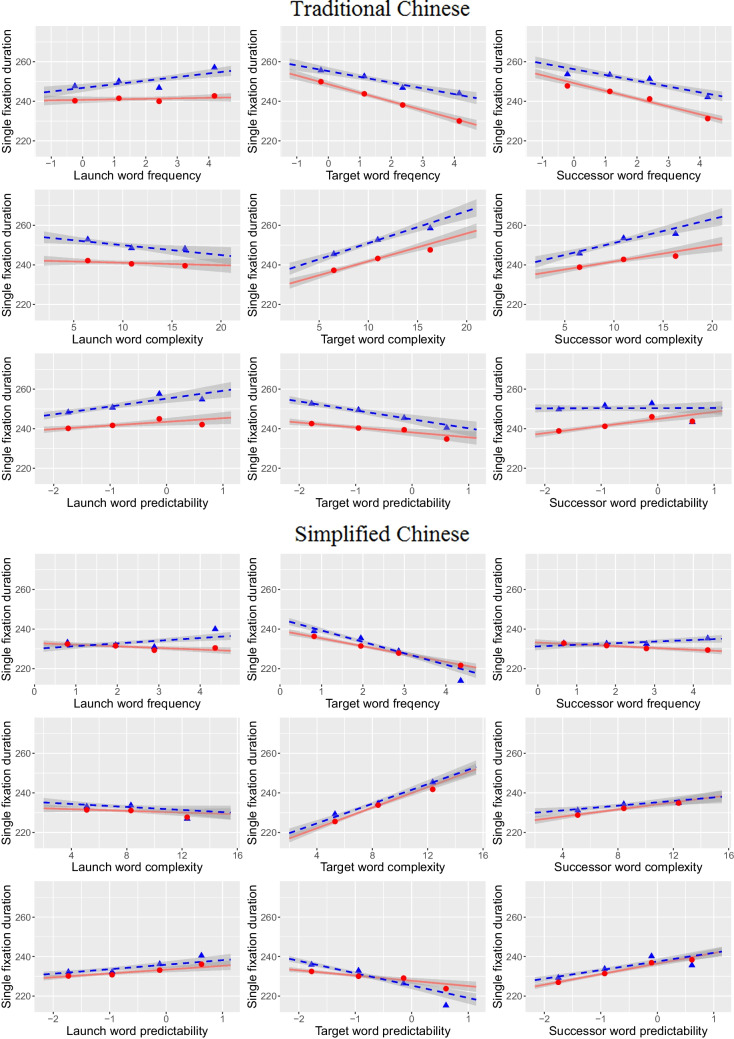
Partial effects (solid lines) of LMMs and zero-order smooths of observed values (dashed lines) of single-fixation duration as a function of launch-word, target-word and upcoming-word properties for TC (upper panel) and SC (lower panel) reading. Shaded bands for partial effects represent 95% CIs based on observation-level LMM residuals

Together with FL analyses reported above, we modeled the SFD data with the traditional focus on effects of properties of word N (i.e., length, frequency, visual complexity, and predictability) and those of its left (word N-1) and right neighbors (word N + 1). Table 3 and Fig. 2 demonstrate strikingly similar patterns of the frequency, complexity, and predictability main effects of words N, N-1, and N + 1 in TC and SC. Overall, the word N-1 (i.e., lag) effects are rather weak, only the N-1 predictability effect was significant with a positive slope, presumably because when highly predictable words N-1 were fixated on more briefly, parafoveal processing of words N was reduced (Yan et al., 2012), leading to increased foveal processing of words N and resulting in longer fixation durations. The word N (i.e., immediate) effects are strong. SFDs on words N were longer for less frequent, visually more complex, and less predictable words N. These effects are canonical and have been found in all writing systems. Finally, the word N + 1 (i.e., successor) effects were also observed. SFDs on words N were longer for less frequent and visually more complex words N + 1, suggesting SC and TC Chinese readers may process part of words N + 1 during their fixations on words N. Replicating previous PSC results, the word N + 1 predictability effect appeared in a direction opposite to the word N predictability effect in SC and TC: Lexical processing of a more predictable word N + 1 is shifted to an earlier stage during fixation on word N and therefore increases its fixation duration. In the meanwhile, because part of lexical processing of word N + 1 has been achieved, when the word is subsequently fixated on, the time required for its immediate processing decreases. The word N + 1 predictability effect in SC and TC appeared in the same direction as in many previous studies (e.g., German: 9 of 9 subsamples reported in Table 1 of Kliegl, 2007; Laubrock & Kliegl, 2015; Spanish: Fernández et al., 2014; English with surprisal as indicator: van Schijndel & Schuler, 2016; van Schijndel & Linzen, 2018), where the effect has been attributed to a predictive rather than parafoveal processing. The effect is opposite to the negative N + 1 predictability effect reported in Angele et al., (2015, 2016) for English sentences. However, aside from using English sentences, Angele et al. used conditional trigram transition probability as their indicator for predictability, not probabilities based on cloze procedure. Ong and Kliegl (2008) showed transition probabilities correlate more highly with frequency than cloze predictability. Interestingly, while the negative word N + 1 frequency effects were stronger in TC, the positive word N + 1 predictability effect was stronger in SC. Perhaps, TC readers are more sensitive to linguistic properties of words irrespective of context, whereas SC readers rely more on contextual memory retrieval. These findings seem to contradict with the findings in single word processing in Tsang et al. (2024). It is possible that how linguistic properties of words influence word recognition may be affected by the nature of the different tasks. Further studies are needed to follow up on this cross-script disparity in Chinese reading in different situations.

**Table 3:** Linear mixed model estimates for word N single-fixation duration

		**TC**			**SC**	
**Fixed effect**	**Est**	**SE**	**t**	**Est**	**SE**	**t**
(Intercept)	5.438	0.021	259.001	5.405	0.017	317.228
l.c	−0.039	0.007	−5.257	−0.028	0.008	−3.447
f.c	−0.018	0.003	−5.339	−0.018	0.004	−4.419
lgs.c	0.040	0.006	6.467	0.064	0.008	7.940
p.c	−0.011	0.004	−2.392	−0.012	0.005	−2.396
l1.c	−0.007	0.006	−1.169	−0.008	0.005	−1.570
f1.c	0.001	0.003	0.524	−0.003	0.002	−1.108
lgs1.c	−0.028	0.005	−0.600	−0.003	0.005	−0.700
p1.c	0.007	0.005	1.538	0.010	0.004	2.370
l2.c	−0.013	0.006	−2.162	0.005	0.005	0.981
f2.c	−0.017	0.003	−6.465	−0.005	0.002	−1.885
lgs2.c	0.023	0.006	4.141	0.018	0.006	3.149
p2.c	0.013	0.004	3.232	0.021	0.004	5.481
**Variance component**		**SD**			**SD**	
Word—(Intercept)		0.036			0.064	
Sent—(Intercept)		0.026			0.029	
Subj—p2.c		0.017			0.017	
Subj—lgs2.c		0.024			0.021	
Subj—p1.c		0.023			0.021	
Subj—f1.c		NA			0.005	
Subj—l1.c		NA			0.009	
Subj—p.c		0.020			0.027	
Subj—lgs.c		0.018			0.018	
Subj—f.c		0.013			0.013	
Subj—l.c		0.024			0.029	
Subj—(Intercept)		0.016			0.128	
Residual		0.306			0.271	
Log-likelihood		−6559.4			−4777.7	
Deviance		13,118.8			9555.5	
AIC		13,116.8			9607.5	
BIC		13,362.8			9827.3	
N		26,097			34,689	

l.c = length of word n, f.c = frequency of word n, lgs.c = log stroke count of word n, p.c = predictability of word n, l1.c = length of word n-1, f1.c = frequency of word n-1, lgs1.c = log stroke count of word n-1, p1.c = predictability of word n-1, l2.c = length of word n + 1, f2.c = frequency of word n + 1, lgs2.c = log stroke count of word n + 1, p2.c = predictability of word n + 1, Subj = subject-related variance component, Word = word-(token-)related variance component, Sent = sentence-related variance component

### Gaze duration

**Fig. 3:**
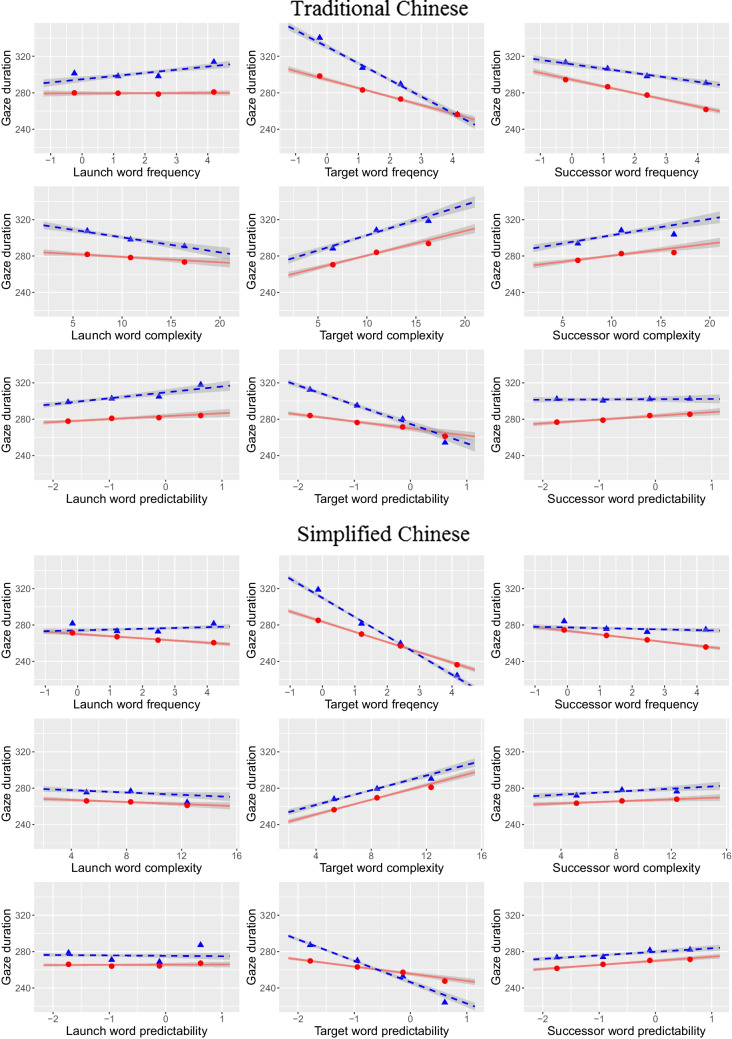
Partial effects (solid lines) of LMMs and zero-order smooths of observed values (dashed lines) of gaze duration as a function of launch-word, target-word and upcoming-word properties for TC (upper panel) and SC (lower panel) reading. Shaded bands for partial effects represent 95% CIs based on observation-level LMM residuals

GD is a widely reported oculomotor measure in reading research. Overall, while first-fixation duration (duration of the first fixation on a word irrespective of the number of fixations) is typically considered to indicate an earlier processing stage, GD may reflect lexical integration processes (e.g., Inhoff, 1984; Inhoff & Radach, 1998). Similar to the SFD results reported above, Table 4 and Fig. 3 demonstrate the frequency, complexity, and predictability effects of words N, N-1, and N + 1 in GD. Once again, TC and SC reading patterns are highly similar. The word N-1 effects were relative weak and the SC readers showed a stronger lag word frequency effect than the TC readers. As expected, the word N effects in GD mirrored those in SFD. Finally, the word N + 1 effects in GD were overall stronger for TC than for SC reading. The readers processed words N longer for less frequent, visually more complex, and more predictable words N + 1.

**Table 4:** Linear mixed model estimates for word N gaze duration

		**TC**			**SC**	
**Fixed effect**	**Est**	**SE**	**t**	**Est**	**SE**	**t**
(Intercept)	5.549	0.026	209.482	5.405	0.019	290.807
l.c	0.070	0.011	6.201	0.104	0.013	8.134
f.c	−0.026	0.004	−6.252	−0.036	0.004	−8.299
lgs.c	0.062	0.009	6.743	0.081	0.011	7.381
p.c	−0.020	0.005	−4.170	−0.022	0.004	−5.448
l1.c	−0.002	0.008	−0.278	−0.010	0.007	−1.517
f1.c	0.000	0.003	0.125	−0.008	0.003	−3.114
lgs1.c	−0.010	0.006	−1.772	−0.011	0.006	−1.866
p1.c	0.010	0.004	2.343	0.004	0.004	0.922
l2.c	−0.021	0.008	−2.623	−0.010	0.007	−1.544
f2.c	−0.026	0.003	−7.865	−0.016	0.003	−5.547
lgs2.c	0.033	0.007	4.504	0.010	0.007	1.357
p2.c	0.013	0.004	3.087	0.016	0.004	4.330
lc:f.c	NA	NA	NA	−0.034	0.005	−6.273
lgs.c:p.c	NA	NA	NA	0.026	0.010	2.654
p.c:p1.c	NA	NA	NA	0.011	0.004	2.687
lgs2.c:p2.c	NA	NA	NA	0.025	0.007	3.733
**Variance component**		**SD**			**SD**	
Word—(Intercept)		0.068			0.085	
Sent—(Intercept)		0.029			0.029	
Subj—lgs2.c		0.031			0.032	
Subj—lgs.c		0.033			0.036	
Subj—l.c		0.053			0.060	
Subj—(Intercept)		0.020			0.142	
Residual		0.404			0.370	
Log-likelihood		−18,357.8			−22,464.7	
Deviance		36,715.6			44,929.3	
AIC		36,755.6			44,977.3	
BIC		36,924.7			45,189.5	
N		34,698			50,968	

l.c = length of word n, f.c = frequency of word n, lgs.c = log stroke count of word n, p.c = predictability of word n, l1.c = length of word n-1, f1.c = frequency of word n-1, lgs1.c = log stroke count of word n-1, p1.c = predictability of word n-1, l2.c = length of word n + 1, f2.c = frequency of word n + 1, lgs2.c = log stroke count of word n + 1, p2.c = predictability of word n + 1, Subj = subject-related variance component, Word = word-(token-)related variance component, Sent = sentence-related variance component

To sum up, the present study established eye movements during the reading of TC sentences. The core patterns of oculomotor behaviors are fundamentally similar between TC and SC skilled adults. The original BSC has been widely used for different research topics such as the perceptual span in Chinese (Pan et al., 2017; Yan et al., 2015), the development of saccade generation in Chinese reading (Yan et al., 2019b), and effects of aging on eye movements (Pan et al., 2024). The BSC-II is well suited for exploring similar research questions and establishing cross-script similarities and disparities in TC and SC. Especially, although TC and SC reading patterns are highly similar, certain visual/linguistic characteristics and the flexibility in reading direction may impose a difference between TC and SC. For instance, from a developmental perspective, visually more complex TC characters may be more difficult to master. However, radicals of some TC characters are more meaningful to memorize than corresponding SC characters, such as the TC character for *love* (愛) has a component for *heart* (心) in the middle of the character, which is lost in the simplified version (SC character: 爱). Additionally, for older adults, visually more complex TC characters may lead to a higher difficulty in visual word recognition and a higher crowding effect, both of which predict a lower reading efficiency and a smaller perceptual span for older TC than SC adults. TC (also Japanese, but not SC) allows flexibility in text orientation, that is, sentences can be written not only in a left-to-right horizontal direction but also in vertical columns from top to bottom. Recently, focusing on this TC characteristic, studies have explored vertical saccade generation (Yan et al., 2019a) and vertical parafoveal processing (Pan et al., 2022b). Yan et al. (2019a) proposed that a commonly observed advantage in horizontal over vertical saccade generation (e.g., Foulsham et al., 2008) is likely caused by overwhelming reading experience in the former direction. To confirm this hypothesis, they demonstrated that Taiwanese readers, who were efficient in text reading in both horizontal and vertical directions, had equivalent reading speeds and fixation-location distributions in the two directions (see also Yan et al., 2024). Pan et al. (2022b) reported that during the vertical reading of TC sentences, Taiwanese readers obtained parafoveal information from upcoming words located underneath the currently fixated one, in the same way as horizontal TC reading (e.g., Tsai et al., 2012). This includes parafoveal extraction of semantic information. However, processing of parafoveal semantic information in TC reading seems to appear later than in SC, probably due to higher visual complexities of TC characters as compared to SC characters. As such, the cross-script comparison between TC and SC is theoretically and practically important and is worth further investigation. In this case, the currently available BSC in TC and SC provide a handy platform for researchers to follow up on the research topics.

## Data Availability

The BSC-II is available publicly from the Open Science Framework from this link: https://osf.io/j9xt4/. The file “BSCII_word_info.xlsx” includes relevant linguistic information for each word in the BSC-II. The file “BSCII.EMD.zip” contains the eye-tracking data from 60 native TC readers as described in Table 1. Finally, “R-scipt.zip” provides scripts for LMM analyses.

## Footnotes

1 Kliegl et al. (2014) reported analyses for an earlier version of TC sentences modeled on the BSC for Taiwanese readers (i.e., the Taipei Sentence Corpus) at the Sixth Chinese Conference on Eye Movements.

2 Word predictability can also be derived from large language models, due to recent development of deep learning based on computational language models (e.g., Berzak & Levy, 2023; Wilcox et al., 2023).
